# Nursing Home Residents and *Enterobacteriaceae* Resistant to Third-Generation Cephalosporins

**DOI:** 10.3201/eid1006.030662

**Published:** 2004-06

**Authors:** Carolyn Sandoval, Stephen D. Walter, Allison McGeer, Andrew E. Simor, Suzanne F. Bradley, Lorraine M. Moss, Mark B. Loeb

**Affiliations:** * McMaster University, Hamilton, Ontario, Canada;; † Mount Sinai Hospital, Toronto, Ontario, Canada;; ‡ Sunnybrook and Women’s College Health Science Centre, Toronto, Ontario, Canada;; § Veterans’ Affairs Health Systems, and University of Michigan Medical School, Ann Arbor, Michigan, USA

**Keywords:** Cephalosporin Resistance, Cephalosporins, Enterobacteriaceae, Enterobacteriaceae Infections, Long-Term Care, Nursing Homes, Risk Factors

## Abstract

Limited data identify the risk factors for infection with *Enterobacteriaceae* resistant to third-generation cephalosporins among residents of long-term-care facilities. Using a nested case-control study design, nursing home residents with clinical isolates of *Enterobacteriaceae* resistant to third-generation cephalosporins were compared to residents with isolates of *Enterobacteriaceae* susceptible to third-generation cephalosporins. Data were collected on antimicrobial drug exposure 10 weeks before detection of the isolates, facility-level demographics, hygiene facilities, and staffing levels. Logistic regression models were built to adjust for confounding variables. Twenty-seven case-residents were identified and compared to 85 controls. Exposure to any cephalosporin (adjusted odds ratio [OR] 4.0, 95% confidence interval [CI] 1.2 to13.6) and log percentage of residents using gastrostomy tubes within the nursing home (adjusted OR 3.9, 95% CI 1.3 to 12.0) were associated with having a clinical isolate resistant to third-generation cephalosporins.

Antimicrobial drug resistance is a concern in nursing homes, facilities where most residents are elderly, frail, and on multiple medications. Gram-negative bacteria resistant to third-generation cephalosporins have emerged as a challenge both in the acute and long-term-care setting (*1**–**9*). These organisms, such as those that produce extended-spectrum β-lactamase (ESBL) or contain the AmpC β-lactamase, can spread rapidly, especially in close living quarters (*1**,**2*).

Identifying modifiable risk factors for acquiring these organisms, such as antimicrobial drug use, can clarify strategies to reduce spread (*1**,**2*). Little is known about whether prior exposure to antimicrobial drugs is a risk factor for *Enterobacteriaceae* resistant to third-generation cephalosporins in the long-term care setting. Wiener et al. reported that prior exposure to ciprofloxacin or trimethoprim-sulfamethoxazole was an independent predictor of colonization with *Escherichia coli* resistant to ceftazidime among nursing home residents (*2*). Molecular analysis of isolates showed that a particular resistance-conferring plasmid appeared frequently, which supports the growing concern that long-term facilities may act as a reservoir for antimicrobial drug–resistant organisms. We previously conducted a prospective, cohort study that examined risk factors for antimicrobial drug resistance in 50 nursing homes in Canada and the United States (*10*). Using these data, we performed a nested case-control analysis to assess whether prior exposure to antimicrobial drugs was a risk factor for infection with *Enterobacteriaceae* that are resistant to third-generation cephalosporins. Staffing characteristics, facilities for hand hygiene, and nursing home resident characteristics were examined as potential risk factors as well.

## Materials and Methods

### Study Design

The methods of the original study from which these data were acquired have been previously published (*10*). Briefly, 50 nursing homes with >100 beds in four provinces (Ontario, Manitoba, Alberta, Saskatchewan) and four states (Minnesota, Michigan, North Dakota, Montana) were enrolled in the study. During a 12-month period, residents treated with systemic antimicrobial drugs were identified, and antimicrobial prescriptions were recorded, including name, dose, and duration. Infection control practitioners from each facility recorded the name and antimicrobial-susceptibility patterns of all clinical bacterial cultures obtained from study residents. Private laboratories or hospital laboratories performed laboratory testing. Susceptibility testing was performed by using methods recommended by the National Committee for Clinical Laboratory Standards (NCCLS). Complete information on all clinical bacterial cultures sent for testing from each home was obtained. These clinical isolates were obtained for diagnostic purposes among residents in the nursing homes. To minimize biased sampling, only cultures sent for signs of suspected clinical infection were included. None of the homes at the time of study collected bacterial specimens from residents for surveillance purposes only. *Enterobacteriaceae* were identified by conventional methods. A survey was performed before the study to ensure that laboratories adhered to NCCLS methods. Infection control practitioners from each facility recorded the name and antimicrobial-susceptibility patterns of all clinical bacterial cultures obtained from study residents. Data recorded for each nursing home included the following: number of resident beds; staffing levels (registered and nonregistered nursing staff hours, healthcare aide hours, infection control practitioner hours, physicians hours, and access to an infectious disease specialist); infrastructure for hygiene facilities (number of showers and tubs per facility and sinks per 100 resident beds); percentage of residents with intravenous catheters, urinary catheters, and gastrostomy tubes; percentage of residents that were either wheelchair or bed bound; and information on recent acute-care stay (new admissions and returns).

### Case Definition

The study population was drawn from the fixed cohort of nursing home residents used in the original study. Only those residents with clinical isolates of *Enterobacteriaceae* resistant or susceptible to third-generation cephalosporins were eligible to be case-residents and controls in this analysis. Case-residents were defined as nursing home residents in whom a clinical *Enterobacteriaceae* isolate resistant to third-generation cephalosporins was detected. For each case-resident, we randomly selected three control-residents from those residents in whom clinical isolates of *Enterobacteriaceae* sensitive to third-generation cephalosporins were obtained. Since repeat isolates may not represent independent events, we assessed only the first clinical isolate obtained from each resident (case-residents and control-residents).

Data were collected at the individual-resident level regarding prior antimicrobial drug use and antimicrobial drug resistance and susceptibility of bacterial isolates. Total antimicrobial drug use, use of any cephalosporins, use of third-generation cephalosporins, use of fluoroquinolone, and use of trimethoprim-sulfamethoxazole were recorded. Covariates were defined as those variables collected at the level of the nursing home, including staffing and patient and facility characteristics.

### Statistical Analysis

We sought to compare antimicrobial drug exposures in nursing home residents from whom *Enterobacteriaceae* resistant to third-generation cephalosporins were isolated to antimicrobial drug exposures in nursing home residents from whom susceptible *Enterobacteriaceae* cultures were obtained. Antimicrobial exposures and nursing home covariates were examined as possible predictors. Log transformations were used when distributions of variables were skewed. All possible predictors were tested for collinearity. Antimicrobial drug exposure was measured in the 10 weeks before resistant bacteria were detected in residents and was compared to antimicrobial drug exposure during a 10-week interval in residents with susceptible organisms. In the absence of any evidence defining an optimal period for assessment of antimicrobial drug exposures, 10 weeks was selected by consensus opinion of five infectious disease specialists with research expertise in the field of antimicrobial drug resistance. All antimicrobial exposures were considered as binary. This strategy was used because all microbial variables, despite log transformation, were highly skewed. Odds ratios were calculated, representing the risk of nursing home residents having antimicrobial drug–resistant clinical isolates compared to antimicrobial drug–susceptible organisms. To assess for the possible effect of clustering for facility-level variables, univariate analyses were also performed with logistic regression with random factors (logistic-binomial model) (Egret 2.031, Cytel Software Corp., Cambridge, MA) and compared with a logistic regression model in which the factors are fixed (SPSS 10.0, SPSS Inc., Chicago, IL). A multivariable model was constructed in which variables with a p value <0.25 and variables representing the various antimicrobial drugs were selected for inclusion, and the final multivariable model was constructed by using a backwards, stepwise approach. All data entry was performed with SAS version 6.0 and 7.0 (SAS Institute, Cary, NC). Analysis was performed by using SPSS 10.0 and Egret 2.031. The Hosmer and Lemeshow test was performed to evaluate the overall fit of the model (*11*). Ethics approval for this study was obtained through McMaster University’s ethics review board.

## Results

Twenty-nine case-residents were identified, and 87 control-residents were initially selected from 26 nursing homes. Because of organizational changes in one nursing home during the course of the study, no covariate data could be obtained. Since such group-level data could not be imputed, all participants selected from this particular home were dropped from the analysis. This included two case-residents and two control-residents. Ignoring the missing group-level variables and including these residents in the analysis on the basis of their individual-level variables made no difference in the estimates subsequently reported (data not shown). The distribution of types of *Enterobacteriaceae* among case-residents and control-residents is shown in Table 1. A greater proportion of *Citrobacter* and *Enterobacter* species were identified in samples from case-residents compared to samples from control-residents (p = 0.01 for each), and a greater proportion of *E. coli* isolates were identified in samples from control-residents compared to samples from case-residents (p = 0.01). Most clinical specimens were isolated from urine samples, and a greater proportion of urine isolates were detected in specimens from control-residents as compared to case-residents (p = 0.05) (Table 2).

**Table 1:** Distribution of *Enterobacteriaceae* organisms isolated from case-patients and controls

Genus	Case-patients (%)	Controls (%)
*Proteus* spp.	7 (26)	17 (20)
*Citrobacter* spp.	6 (22)	4 (5)
*Enterobacter* spp.	5 (18)	2 (2)
*Escherichia coli*	4 (15)	47 (55)
*Klebsiella* spp.	3 (11)	11 (13)
*Morganella* spp.	1 (4)	4 (5)
*Serratia marcescens*	1 (4)	0 (0)
Total	27	85

**Table 2:** Distribution of sites from which *Enterobacteriaceae* were isolated

Site	Case-patients (%)	Controls (%)
Urine	20 (74)	77 (91)
Wound	3 (11)	2 (2)
Eye	3 (11)	2 (2)
Skin	1 (4)	3 (4)
Sputum	0 (0)	1 (1)
Total	27	85

Univariate analyses of individual-level exposures and facility-level exposures are shown in Tables 3 and 4. The following variables were considered in the multivariable model: prior exposure to any antimicrobial drug, to a cephalosporin, to a third-generation cephalosporin, to a fluoroquinolone, or to trimethoprim-sulfamethoxazole; the log number of primary care physician hours per 100 resident beds; whether an infectious disease physician was on staff; the number of new admissions from acute care hospitals within the last year per 100 resident beds; and the log percentage of residents in the nursing home with a gastrostomy tube.

**Table 3:** Univariate logistic regression analyses of individual-level variables with clinical isolate of *Enterobacteriaceae* resistant to third-generation cephalosporins as the dependent variable

Variables^a^	Case-patients (%)	Controls (%)	OR (95% CI)^b^	p value
Any antimicrobial drug	13 (48)	27 (32)	2.0 (0.8 to 4.8)	0.125
Any cephalosporin	8 (30)	6 (7)	5.5 (0.1 to 0.6)	0.004
Third-generation cephalosporin	2 (7)	2 (2)	3.3 (0.04 to 2.2)	0.242
Fluoroquinolone	3 (11)	7 (8)	1.4 (0.1 to 3.0)	0.649
Trimethoprim/sulfamethoxazole	4 (15)	5 (6)	2.8 (0.1 to 1.4)	0.150

^a^10 weeks before date of confirmed Enterobacteriaceae infection. ^b^OR, odds ratio; CI, confidence interval.

**Table 4:** Univariate logistic regression analyses of nursing home–level variables with clinical isolate of *Enterobacteriaceae* resistant to third-generation cephalosporins as the dependent variable^a^

Variables	OR (95% CI)	p value
>200 beds (no.)	0.85 (0.35 to 2.05)	0.718
Nursing units per 100 beds (log no.)	4.09 (0.25 to 66.45)	0.322
RN FTE h (log no.)	1.21 (0.18 to 8.31)	0.846
NA FTE h (log no.)	1.91 (0.44 to 8.43)	0.391
Healthcare aide FTE h (log no.)	0.99 (0.95 to 1.02)	0.356
ICP FTE h (log no.)	0.91 (0.19 to 4.43)	0.910
Primary care physician FTE h (log no.)	0.28 (0.08 to 0.91)	0.034
Infectious disease specialist on staff	0.40 (0.16 to 1.03)	0.057
Tubs/showers per 100 beds/facility (log no.)	2.25 (0.48 to 10.48)	0.303
Sinks per 100 beds (no.)	1.00 (0.99 to 1.01)	0.965
New admissions from to acute care prior y/100 beds (no.)	1.00 (0.99 to 1.01)	0.205
Residents readmitted acute care hospitals prior y/100 beds (no.)	1.01 (0.99 to 1.03)	0.481
Residents with urinary catheter (%)	1.01 (0.90 to 1.13)	0.923
Residents with intravenous catheter (%)	1.64 (0.68 to 3.9)	0.272
Residents with gastrostomy tube (log %)	5.02 (1.72 to 14.70)	0.003
>50% residents unable to ambulate (no.)	1.34 (0.54 to 3.31)	0.533

^a^OR, odds ratio; CI, confidence interval; FTE, full-time equivalent; RN, registered nurse; NA, nursing aide; ICP, infection control practitioner.

The only variables that remained significant after multivariable modeling were prior use of any cephalosporin (odds ratio [OR] 4.0, 95% confidence interval [CI] 1.2 to 13.6, p = 0.029), and log percentage of residents with a gastrostomy tube (OR 3.9, 95% CI 1.3 to 12.0, p = 0.016). The p value obtained for the Hosmer and Lemeshow test was 0.138, which suggests that the overall fit of this model is reasonable.

## Discussion

*Enterobacteriaceae* infections resistant to cephalosporins are of concern in long-term care facilities and in the acute-care setting (*1**–**9*). Patients with infections resistant to third-generation cephalosporins have been reported to have had longer hospital stays, higher death rates, and greater hospital costs than patients whose infections are susceptible to third-generation cephalosporins (*4*). A survey of infection control practitioners in Ontario showed that no standard approach exists to dealing with ESBL-producing *E. coli* and *Klebsiella* spp. in long-term-care facilities (*12*). Reservoirs of resistant *Enterobacteriaceae* species will continue to emerge in this setting despite implementation of control measures.

Our findings show that recent exposure to any cephalosporin is associated with the isolation of third-generation cephalosporin–resistant *Enterobacteriaceae* in nursing home residents. Knowledge of previous exposure may help physicians anticipate this particular pattern of resistance.

Few studies have assessed risk factors for *Enterobacteriaceae* resistance to cephalosporins in long-term-care facilities. Weiner et al. described an outbreak of ceftazidime-resistant *E. coli* infections in Chicago nursing homes (*2*). Those researchers conducted a case-control study and found that ciprofloxacin or trimethoprim-sulfamethoxazole exposure was associated with ceftazidime-resistant *E. coli* in nursing home residents. Cephalosporin treatment may have been given after fluoroquinolone resistance was detected, and this treatment may be linked to cephalosporin resistance found in their study. Muder et al. evaluated modifiable risk factors for antimicrobial drug–resistant *Enterobacteriaceae* infection among patients from a long-term Veterans Affairs facility in Pittsburgh (*13*). In this case-control study, patient debility, age, coexisting conditions, and prior antimicrobial therapy were examined as risk factors associated with multidrug-resistant *Enterobacteriaceae* infections. Case-patients were identified as having an *Enterobacteriaceae* infection resistant to two of the following groups of antimicrobial drugs: piperacillin, third-generation cephalosporins, or gentamicin. Only a pressure ulcer [OR 12.2, 95% CI 3.3 to 44.2] and prior ampicillin therapy [OR 13.7, 95% CI 2.2 to 84.0] were associated with resistant *Enterobacteriaceae* infection. In contrast, we did not assess individual-level covariates. No association was found between prior cephalosporin therapy and a multiple-resistant infection, but whether prior cephalosporin therapy and the acquisition of cephalosporin-resistant infections are linked is unknown. A similar study performed previously by this group identified exposure to ciprofloxacin as a risk factor for the acquisition of ciprofloxacin-resistant gram-negative bacteria (*14*). Our findings confirm that patient debility and prior antimicrobial drug therapy are associated with acquisition of resistant bacteria.

In the acute-care setting, similar risk factors for the acquisition of broad-spectrum cephalosporin-resistant *Enterobacteriaceae* have been recognized (*15**–**19*). The risk of acquiring multidrug-resistant bacterial infection has been reported as increasing in hospitalized patients with prior third-generation cephalosporin therapy (*6**,**18**,**19*). A hospital-based case-control study performed in Argentina examined risk factors for ceftazidime-resistant *K. pneumoniae*. Prior antimicrobial drug use was associated with acquisition of this type of resistant infection [adjusted OR 6.21, 95% CI 1.20 to 32.01]. Other risk factors found were prior use of ciprofloxacin, nosocomial infection, and hospitalization stay >6 days (*15*). Bisson et al. also looked at risk factors for acquisition of ESBL-producing *Klebsiella* spp. and *E. coli* in which patients with this resistance pattern were compared to patients with no culture exhibiting this resistance pattern (*16*). Only length of hospitalization stay was associated with colonization with ESBL–producing *E. coli* and *Klebsiella* spp. [adjusted OR 1.11, 95% CI, 1.02 to 1.21]. Prior receipt of antimicrobial therapy was not associated with colonization in these patients, but none were exposed to third-generation cephalosporins. Since our study was set in long-term-care facilities and because individual measurements were not done, length of stay was not assessed. Lin et al. performed a case-control study evaluating risk factors for ESBL–producing *K. pneumoniae* among hospital patients in Taiwan (*17*). Prior use of ceftazidime was found to be associated with ESBL-producing *K. pneumoniae* infection compared to non-ESBL-producing *K. pneumoniae* infection. A case-control study by Bonomo et al. did not find an association between fecal colonization of cefotaxime-resistant gram-negative bacilli and antimicrobial drug use 4 weeks before admission to the hospital (*20*).

Comparison of antimicrobial drug-sensitive organisms to antimicrobial drug-resistant organisms cannot infer absolute risk for exposure to that antimicrobial drug (*21**,**22*). However, Lipsitch has discussed several advantages of calculating conditional OR (OR_c_) versus simple OR (OR_s_) when interpreting associations between antimicrobial drug use and resistant organisms (*23*). OR_s_ determines a patient’s individual risk of acquiring a resistant infection after receiving treatment compared to not getting an infection after receiving treatment. OR_c_ determines the risk of a resistant infection compared to the risk of a nonresistant infection, which allows for insight into whether a suspect infection in a patient is more likely to be resistant to a particular antimicrobial drug compared to someone who has not been treated (*23*). Widespread treatment with antimicrobial drugs promotes the eradication of antimicrobial-susceptible organisms but also confers a selective advantage for developing antimicrobial-resistant organisms. Measuring OR_c_ may even be more useful for clinicians than knowing the absolute risk because it gives information about the community-level risk of having a resistant organism compared to a susceptible organism in persons with a particular recent antimicrobial drug history (*23*). Making this comparison is useful for clinicians who are evaluating the odds of a patient’s being infected with a resistant organism.

In addition to antimicrobial drug exposure, the percentage of residents with gastrostomy tubes in the home was also found to predict *Enterobacteriaceae* infection resistant to third-generation cephalosporins. In this analysis, percentage of gastrostomy tubes among home residents served as a proxy for generalized debility within each nursing home, and our findings suggest that more debilitated patients may be more predisposed to acquiring resistant organisms. Debilitated patients are most likely to harbor resistant organisms (*24*). Since we do not know the strain of resistant bacteria applicable to each patient, we cannot infer with certainty that a gastrostomy tube in a nursing home resident is a risk factor for this type of resistant infection. However, Weiner et al. identified gastrostomy tube use at the patient-level as a risk factor for acquiring resistant *Enterobacteriaceae* (*2*). Infections directly associated with gastrostomy tube contamination, resulting in antimicrobial treatment or hospitalization (*25**,**26*), have also been reported,. However, since gastrostomy tube sites were not systematically tested in this study, this finding cannot be inferred.

Strengths of this study are that residents were drawn from a large number of nursing homes in both Canada and the United States, exposures at both the individual and aggregate levels were examined, and a wide range of variables were considered, including staffing and infrastructure for hand hygiene. Our study has some limitations, including the following: the date of actual acquisition of resistant bacteria is not known; we only examined clinical isolates, obtained for diagnostic purposes, which may have led to a sampling bias; and individual level coexisting conditions were not measured. Given the large size of the original study, measuring coexisting conditions was not feasible. Another limitation is that facilities with higher levels of resistant bacteria among residents may obtain more clinical specimens (or vice versa), which may have led to bias in the sample used. Cross-infection within a facility could not be assessed because of lack of molecular typing of isolates. Admissions and readmissions to acute-care facilities within the last year were not identified as risk factors for infection with *Enterobacteriaceae* resistant to third-generation cephalosporins. The 12-month period may have been too long, but a significant association may have been identified with a 3-month period. In conclusion, prior exposure to cephalosporins and an increased use of gastrostomy tubes among nursing home residents predicts for infection with *Enterobacteriaceae* resistance to third-generation cephalosporins.
